# Characteristics of the tumor microenvironment and potential immunotherapy strategies in renal cell carcinoma

**DOI:** 10.3389/fimmu.2025.1643533

**Published:** 2025-09-02

**Authors:** Hui Wen, Shi Zheng, Xiaoqin Zhu, Ling Wang, Dongping Chen

**Affiliations:** ^1^ Department of Nephrology, Shuguang Hospital Affiliated to Shanghai University of Traditional Chinese Medicine, Shanghai, China; ^2^ Traditional Chinese Medicine (TCM) Institute of Kidney Disease of Shanghai University of Traditional Chinese Medicine, Shanghai, China; ^3^ Key Laboratory of Liver and Kidney Diseases, Ministry of Education, Shanghai, China; ^4^ Shanghai Key Laboratory of Traditional Chinese Clinical Medicine, Shanghai University of Traditional Chinese Medicine, Shanghai, China; ^5^ Department of Gastroenterology, The Affiliated Hospital of Southwest Medical University, Luzhou, China; ^6^ Department of Hematology, Shanghai Shuguang Hospital Affiliated to Shanghai University of Traditional Chinese Medicine, Shanghai, China; ^7^ Department of Nephrology, Shanghai Tenth People’s Hospital Affiliated to Tongji University, Shanghai, China

**Keywords:** renal cell carcinoma, tumor microenvironment, immunosuppressive cells, biomarkers, immunotherapy, combined targeted/immunotherapy

## Abstract

Renal cell carcinoma (RCC) is a highly vascularized and immunogenic malignancy with a complex tumor microenvironment (TME) that shapes disease progression and therapeutic resistance. Despite advances in immune checkpoint inhibitors (ICIs) and targeted therapies, clinical responses remain heterogeneous, underscoring the need for a deeper understanding of RCC immunobiology. This review comprehensively examines the immunosuppressive TME of RCC, emphasizing the roles of cytotoxic and immunosuppressive immune cells, carcinoma-associated fibroblasts (CAFs), abnormal vasculature, and extracellular matrix (ECM) remodeling in fostering immune evasion. This review summarized emerging biomarkers—including PD-L1 expression, tumor mutational burden (TMB), gene mutations, and immune-based subtypes—that may predict ICI response. Furthermore, we evaluate current immunotherapeutic strategies, such as ICIs, combination therapies, and novel approaches targeting immunosuppressive cells and metabolic pathways. While combination therapies have improved outcomes, challenges like toxicity and resistance persist, necessitating biomarker-driven patient stratification and optimized treatment sequencing. Future directions should focus on deciphering TME heterogeneity and developing precision immunotherapy strategies to enhance clinical efficacy in RCC.

## 1 Introduction

Renal cell carcinoma (RCC), a lethal genitourinary tumor originating from renal tubular epithelial cells, ranks among the top fifteen cancers globally (1). It exhibits a 30–40% mortality rate, with higher prevalence in males. Risk factors include obesity, hypertension, smoking, and chronic kidney disease (2). Early-stage RCC is often asymptomatic; however, advances in CT, MRI, PET-CT, and genetic testing have improved detection, with over 60% of cases diagnosed incidentally. While early-stage patients benefit from surgery, 30% present with metastasis at diagnosis. Post-surgical recurrence occurs in 30–40% of advanced cases, and 50% develop distant metastases, leading to poor prognosis (3, 4).

Clear cell RCC (ccRCC) is the most common subtype and dominates metastatic RCC (mRCC) pathology. Due to RCC’s resistance to radiation/chemotherapy, targeted therapy has been the first-line treatment, though drug resistance remains inevitable (5, 6). Immune checkpoint inhibitors (ICI) show efficacy, but only a subset of patients show response, potentially due to the immunosuppressive tumor microenvironment (TME) (7, 8). RCC TME features extensive immune infiltration, vascularity, and fibrosis, enabling immunotherapy but also influencing treatment resistance via complex interactions (9, 10). Recent therapeutic strategies for advanced RCC have evolved from targeted therapy to combined targeted/immunotherapy approaches (11, 12). This review discusses RCC TME crosstalk, clinical immunotherapy progress, and emerging TME-based biomarkers/therapeutic targets.

## 2 Characteristics of the TME in renal cell carcinoma

### 2.1 Cytotoxic immune cells

CD8^+^ T cells infiltrating the RCC TME frequently exhibit high expression of inhibitory checkpoint receptors—including programmed death-1 (PD-1), cytotoxic T-lymphocyte-associated protein 4 (CTLA-4), and T cell immunoglobulin and mucin domain-containing protein 3 (Tim-3)—alongside low levels of proliferative markers such as Ki-67, suggesting a state of dysfunction and exhaustion (13, 14). In a meta-analysis of 124 studies, Fridman et al. (15) reported that, unlike in most solid tumors, CD8^+^ T cell infiltration in RCC correlates with poorer prognosis. While the underlying mechanism remains unclear, one hypothesis is that prolonged exposure to immunosuppressive cells and factors within the RCC TME impairs CD8^+^ T cells’ ability to recognize antigens, proliferate, and secrete interleukin-2 (IL-2), ultimately abrogating their cytotoxic functions (16). Recent mechanistic studies indicate that chronic antigen stimulation activates NFAT in the absence of AP−1, which drives the transcription of TOX and WNK1, committing CD8^+^ T cells to an exhausted phenotype (17–19). In RCC, tumor-derived PD-L1 binds PD-1 on CD8^+^ T cells, recruiting SLC11A1 and inactivating ZAP70 and PI3K/AKT signaling, while CTLA−4 competes for B7 ligands on APCs to prevent costimulation (20–24). Additionally, Tim−3–Galectin−9 interactions promote Batf expression, further enforcing the exhausted transcriptional program (25). These events converge to reduce granzyme B production, IFN-γ secretion, and proliferative capacity. Single-cell RNA-seq studies in melanoma and RCC now reveal distinct subsets of exhausted T cells, characterized by high expression of PD−1, TOX, and CXCL13, suggesting specialized niches where these exhausted cells localize (26, 27). Therefore, the functional status of CD8^+^ T cells is pivotal in determining both patient prognosis and the efficacy of immunotherapies. Recent advances in single-cell RNA sequencing (scRNA-seq) have enabled detailed profiling of exhausted CD8^+^ T cells, and this technology has already been applied successfully in melanoma studies (28). Implementing scRNA-seq to characterize RCC-specific TME features may help elucidate the long-observed inverse association between CD8^+^ T cell infiltration and clinical outcomes in RCC (29).

### 2.2 Natural killer cells and immunosuppressive cells

NK cells are another major cytotoxic population capable of mediating anti-tumor immunity through perforin and interferon-γ (IFN-γ) release without prior sensitization (30). Remark et al. (31) demonstrated a positive correlation between NK cell infiltration and favorable prognosis in RCC. However, soluble cytokines, membrane-bound ligands, and TGF-β-enriched exosomes derived from tumor cells and immunosuppressive cells can inhibit NK cell degranulation and cytotoxicity (32–34). In addition to soluble TGF−β, RCC-derived exosomes carry TGF−β and immunomodulatory miRNAs (miR−23a, miR−146a), which are internalized by NK cells and lead to the downregulation of activating receptors such as NKG2D, NKp30, and NKp44 (35–38). This receptor loss reduces their ability to recognize and lyse tumor cells. Furthermore, CAFs secrete abundant prostaglandin E_2_ (PGE_2_), which acts on EP2/EP4 receptors expressed by NK cells (39, 40). Engagement of these receptors triggers the cAMP–PKA–CREB signaling cascade, suppressing the transcription of genes involved in cytotoxic granule formation and IFN−γ production (41–43). The net effect is impaired NK cell proliferation, decreased granule exocytosis, and weakened target cell killing capacity (44). These mechanisms, combined with other immunosuppressive metabolites (adenosine), synergistically dampen NK cell cytotoxicity within the RCC TME (45). Mechanistically, TGF−β binds TGFβRII on NK cells, activating SMAD2/3, which downregulates NKG2D and perforin expression; tumor-derived adenosine acts via A2A receptors to activate PKA signaling, suppressing NK metabolism and granule release (46–48). As a result, the functional capacity of tumor-infiltrating NK cells is often compromised. Therefore, strategies aimed at restoring NK cell activity are critical for enhancing the efficacy of ICIs in RCC (49).

Regulatory T cells (Tregs), a CD4^+^ T cell subset with immunosuppressive function, are essential for immune homeostasis but promote immune evasion in the RCC TME (50). Tumor and stromal cells secrete IL-10, IL-23, TGF-β, adenosine, and adhesion molecules to recruit Tregs, which suppress CD8^+^ T cells via TGF-β, IL-10, and IL-35 (51, 52). Although associated with poor prognosis, the precise role of Tregs in RCC remains unclear and requires further elucidation (53). Tumor-associated macrophages (TAMs), the dominant myeloid population in RCC, polarize into pro-inflammatory M1 and immunosuppressive M2 phenotypes (54). Elevated M2 or M2/M1 ratios correlate with poor outcomes (55). CSF1/CSF1R and IL−4/STAT6 signaling are major inducers of M2 polarization (56, 57). M2 TAMs secrete IL-10, CCL17/22, and VEGF, while activating PI3K/AKT and STAT3 pathways in tumor cells, which promotes proliferation and immune evasion (58–60). RCC-derived M-CSF promotes M2 polarization, comprising up to 20.9% of immune cells (61). M2-TAMs inhibit CD8^+^ T cell cytotoxicity, recruit suppressive cells, and remodel the ECM via MMPs, aiding invasion and metastasis (62). Chevrier et al. identified 17 TAM states, linking CD38^+^M5 TAMs to T cell exhaustion and Tregs, while high M11/M13 and low M5 TAM levels predicted shorter progression-free survival, suggesting therapeutic potential in TAM modulation. Myeloid-derived suppressor cells (MDSCs) inhibit CD8^+^ T cells through PD-L1 expression, ARG1-mediated amino acid depletion, and ADAM17-dependent T cell trafficking. MDSCs also promote immunosuppressive ECM remodeling via MMPs and iNOS (63, 64). ARG1 depletes arginine, limiting TCR ζ-chain expression; iNOS-derived NO leads to nitration of TCR complexes, impairing signal transduction, while NF-κB signaling within MDSCs maintains their suppressive function (65–67). Tie2-expressing monocytes (TEMs) facilitate angiogenesis and RCC progression (68). Neutrophil proteases also remodel the ECM via PAD4-mediated chromatin decondensation, promote invasion, induce T cell exclusion/exhaustion, and contribute to TKI resistance and poor prognosis in RCC (69).

### 2.3 Carcinoma-associated fibroblasts

CAFs, the most abundant stromal cell type in RCC, are central to tumor growth, metastasis, drug resistance, and immune evasion (70, 71). Under hypoxia and oxidative stress, tumor cells secrete TGF-β, IL-6, and platelet-derived growth factor (PDGF), activating CAF precursors, which upregulate fibroblast activation protein (FAP) (72). Activated CAFs stimulate pro-inflammatory signaling pathways such as STAT3 and NF-κB, and secrete hepatocyte growth factor (HGF), epidermal growth factor (EGF), and IL-6 to recruit Treg and activate immunosuppressive cells (73–75). Through secretion of TGF−β and ARG2, CAFs induce M2 polarization of TAMs and expansion of Tregs, while CXCL12 produced by CAFs engages CXCR4 on T cells, forming a “chemokine barrier” that excludes CD8^+^ T cells from tumor nests (76, 77). CAFs can also directly inhibit cytotoxic immune cells via TGF-β and ARG2 secretion (78). As “architects” of the TME, CAFs produce ECM components and facilitate tumor progression and metastasis (79, 80). The immunosuppressive nature of CAFs underlies the poor responsiveness of fibrotic tumors to therapy, yet their ubiquity offers multiple therapeutic targets (81, 82). Although anti-CAF therapies have shown promise in breast and pancreatic cancers, CAF heterogeneity across tumor types necessitates further investigation into RCC-specific CAF-targeting strategies (83).

### 2.4 Vascular endothelial cells

RCC is among the most vascularized tumors, a feature strongly associated with early biallelic inactivation of the tumor suppressor gene von Hippel–Lindau (VHL) (84). VHL negatively regulates hypoxia-inducible factor (HIF), and its loss leads to HIF accumulation and subsequent overproduction of vascular endothelial growth factor (VEGF), promoting tumor angiogenesis (85). VEGF binds VEGFR2 on endothelial cells, activating PI3K–AKT and MAPK/ERK pathways to promote angiogenesis. The resulting abnormal vessels express FasL and downregulate adhesion molecules (ICAM-1, VCAM-1), creating a physical and biochemical barrier to immune (86–88). Abnormal vasculature impairs perfusion, leading to hypoxia, acidosis, and reduced drug penetration. These conditions further induce immunosuppressive factors such as TGF-β, VEGF, and adenosine, and downregulate endothelial adhesion molecules, impeding immune cell adhesion, trafficking, and infiltration (89). While microvascular density serves as a prognostic indicator in cancers such as oral cancer, its prognostic value in RCC remains controversial due to variability in vascular morphology and differentiation (90, 91). Moreover, endothelial cells in RCC express high levels of indoleamine 2,3-dioxygenase (IDO) under IFN-γ stimulation, triggering tryptophan catabolism via the kynurenine pathway. Kynurenine activates aryl hydrocarbon receptor (AHR) in T cells, inducing FOXP3 expression and generating Tregs, further promoting immunosuppression (92).

### 2.5 Extracellular matrix and soluble factors

RCC progression involves extensive ECM deposition, providing structural support, biomechanical signaling, and regulation of cell behavior (93). The ECM, primarily secreted by CAFs, consists of collagens, laminins, glycoproteins, fibronectin, proteoglycans, and polysaccharides (94). Matrix remodeling is mediated by enzymes such as MMP2/9 and lysyl oxidase (LOX), which are activated by TGF−β and hypoxia (HIF−1α). These pathways stiffen the ECM and impair immune cell infiltration (95–97). TAMs, MDSCs, and CAFs secrete transglutaminases and lysyl oxidase, remodeling the ECM to induce collagen rearrangement, matrix stiffening, and reduced permeability, forming a barrier against cytotoxic immune infiltration (98). ECM remodeling also causes mechanical stress, impairing vascular function and promoting immune suppression (99). The remodeled ECM harbors abundant soluble mediators that facilitate bidirectional communication between tumor epithelial and stromal compartments, thereby promoting RCC invasion and metastasis (100, 101). Hypoxia and necrosis in rapidly growing tumors trigger the release of CSF-1, G-CSF, TGF-β, and chemokines (CCL2/3/4/7), recruiting myeloid cells (102–105). These cells, in turn, secrete VEGF, EGF, HGF, PDGF, CXCL12, and IL-8 to sustain tumor growth, angiogenesis, and immune infiltration (106, 107). In addition to amino acid depletion via iNOS and arginase-1, metabolic reprogramming driven by HIF signaling profoundly affects the immunosuppressive milieu (108, 109). RCC cells preferentially undergo aerobic glycolysis, resulting in excess lactate production and extracellular acidification (110). Elevated lactate concentrations reduce the glycolytic capacity of CD8^+^ T cells, suppress mTOR signaling, and promote a state of metabolic exhaustion (111). Lactate also enhances histone lactylation, which epigenetically upregulates PD−1 expression, thereby intensifying T cell dysfunction in synergy with PD−1/PD−L1 signaling (112, 113). Moreover, lactate accumulation favors the expansion of Tregs and M2-polarized macrophages, creating a positive feedback loop that reinforces immune evasion (114, 115). These mechanisms intersect with IDO- and arginase-mediated nutrient depletion, collectively dampening T cell activation and effector function within the RCC TME. Depletion of specific soluble factors also plays a critical role in immune evasion. Tumor cells consume large quantities of glucose and glutamine, the latter being essential for T-bet expression and CD4^+^ T cell differentiation (116). Enzymes such as iNOS and ARG1 from myeloid cells and CAFs and IDO from endothelial cells deplete essential amino acids and generate toxic metabolites, directly impairing T cell function (117) (**Table 1**).

**Table 1:** Immunosuppressive components of the RCC tumor microenvironment and their roles in immune evasion.

Component	Features	Immune Mechanisms	Clinical Impact	Therapeutic Targets
Cytotoxic CD8^+^ T cells	High PD-1, CTLA-4, Tim-3 expression; low Ki-67; exhausted phenotype	Impaired antigen recognition, proliferation, and IL-2 secretion due to chronic immunosuppressive signals	Meta-analysis shows infiltration correlates with poor prognosis; functional status determines immunotherapy response	PD-1/CTLA-4 blockade, scRNA-seq-guided reinvigoration strategies
NK cells	Mediate cytotoxicity via perforin/IFN-γ; inhibited by TGF-β, exosomes, and soluble ligands	Degranulation and cytotoxicity suppressed by TME-derived factors	Infiltration associated with favorable prognosis, but function often compromised	Cytokine priming (IL-15), TGF-β inhibition, exosome blockade
Tregs	CD4^+^ subset recruited via IL-10, TGF-β, adenosine; suppress via IL-10/IL-35/TGF-β	Direct inhibition of CD8^+^ T cells; promotion of T cell exhaustion	High infiltration linked to poor prognosis, but role in RCC remains controversial	Depletion (anti-CD25), TGF-β/IL-10 pathway inhibition
M2-TAMs	Dominant myeloid population (up to 20.9% of immune cells); polarized by M-CSF	ECM remodeling (MMPs), CD8^+^ T cell inhibition, recruitment of suppressive cells (Tregs, MDSCs)	High M2/M1 ratio correlates with poor outcomes; CD38^+^M5 subset linked to T cell exhaustion	CSF-1R inhibition, repolarization to M1 (TLR agonists)
MDSCs	Express PD-L1, ARG1, iNOS; secrete MMPs	Amino acid depletion (ARG1), T cell trafficking inhibition (ADAM17), ECM remodeling	Promote TKI resistance; correlate with advanced disease	Entinostat (ARG1/iNOS suppression), CXCR4 antagonists (AMD3100)
CAFs	Activated by TGF-β/IL-6/PDGF; secrete HGF, EGF, IL-6, ECM components	Direct T cell suppression (TGF-β, ARG2); ECM stiffening; recruitment of immunosuppressive cells	Fibrosis associated with therapy resistance; FAP expression predicts invasiveness	FAP-targeted therapies (CAR-T, vaccines), STAT3/NF-κB inhibition
Abnormal Vasculature	Driven by VHL-HIF-VEGF axis; dysfunctional perfusion	Hypoxia-induced TGF-β/VEGF/adenosine; impaired immune cell adhesion/trafficking	Microvascular density prognostic value debated; IDO^+^ endothelial cells promote immune evasion	VEGF inhibitors (axitinib), IDO blockade (epacadostat)
ECM Remodeling	Collagens, fibronectin, proteoglycans stiffened by LOX/transglutaminases (from CAFs/TAMs/MDSCs)	Physical barrier to immune infiltration; mechanical stress impairs vascular function	Correlates with advanced stage and metastasis	LOX/MMP inhibitors, mechanotherapy (YAP/TAK1 targeting)

## 3 Biomarkers for immunotherapy

### 3.1 PD-L1 expression and tumor-infiltrating lymphocytes

Numerous clinical trials in RCC have reported that only a small subset of patients can achieve complete response and tolerate long-term immunotherapy, while the majority experience disease progression (118, 119). Therefore, the identification of reliable biomarkers capable of predicting immunotherapeutic response is critical for selecting patients most likely to benefit from such treatments (120, 121). Tumor PD-L1 expression is the most widely used biomarker for predicting responses to PD-1/PD-L1 blockade therapy and one of the earliest predictive indicators studied in RCC (122). Although high PD-L1 expression in RCC tissues has been associated with poor prognosis, PD-L1 alone is insufficient to predict therapeutic efficacy (123). Stenzel et al. (124) demonstrated that tumor tissues from patients with ccRCC who responded favorably to ICIs exhibited significantly higher CD8^+^ T cell infiltration and PD-L1 positivity compared to non-responders. ICIs can reinvigorate pre-existing Th1 cells within the TME, enabling cytotoxic responses against tumor cells (125, 126). This seemingly paradoxical relationship between PD-L1 expression, poor prognosis, and ICI responsiveness may reflect both the spatial heterogeneity of PD-L1 expression in tumor cells and its dynamic regulation: inducible PD-L1 upregulation by IFN−γ released during an active anti-tumor immune response versus constitutive PD-L1 expression driven by HIF−1α in hypoxic regions (127). These mechanisms highlight that PD-L1 expression must be interpreted in the context of the tumor microenvironment and cellular localization (128, 129). Therefore, patients with this immune phenotype are more likely to benefit from ICI therapy.

### 3.2 Gene mutations

TMB and microsatellite instability (MSI) are well-established predictive biomarkers for ICI efficacy across several malignancies (130, 131). It is generally accepted that tumor-specific neoantigens generated by somatic mutations facilitate immune infiltration, a prerequisite for ICI responsiveness (132, 133). Despite the high immune infiltration in RCC, TMB levels are significantly lower compared to other immunogenic tumors such as lung adenocarcinoma and melanoma (134). A pan-cancer analysis of 19 malignancies by Turajlic et al. (135) using The Cancer Genome Atlas (TCGA) data revealed that RCC harbors the highest frequency and count of insertion or deletion (indel) mutations—over twice the average observed in other cancers. Further RNA sequencing of 329 RCC samples confirmed that indel mutations are associated with heightened immunogenicity, suggesting that indels may serve as superior predictive biomarkers compared to TMB in RCC. Over 90% of sporadic ccRCC cases involve chromosomal translocations at 3p, leading to frequent mutations in VHL, PBRM1, BAP1, and SETD2. Consequently, RCC is considered a disease defined by genomic rearrangements (136). Messai et al. (137) reported a positive correlation between VHL mutations and PD-L1 expression in ccRCC, which may influence patient responses to immunotherapy. In a prospective study, Miao et al. (138) performed whole-exome sequencing on tumor tissues from 35 untreated mRCC patients and found that loss-of-function mutations in *PBRM1* were associated with enhanced responsiveness to ICIs, a finding subsequently validated in independent cohorts. A retrospective analysis of the CheckMate 025 trial further demonstrated that *PBRM1*-mutant RCC patients experienced significantly prolonged progression-free survival (PFS) and overall survival (OS) following anti-PD-1 therapy (139). Mechanistically, loss of PBRM1 disrupts the SWI/SNF chromatin remodeling complex, leading to changes in nucleosome positioning and transcriptional accessibility of interferon-stimulated genes (140). This epigenetic reprogramming can activate the STING–type I interferon pathway, increasing tumor immunogenicity and chemokine production (CXCL10, CCL5), thereby enhancing dendritic cell recruitment and T cell priming (141, 142). Additionally, PBRM1 deficiency has been associated with increased expression of MHC II molecules and components of the antigen-processing machinery, potentially improving tumor antigen presentation and amplifying CD8^+^ T cell responses (143, 144).

### 3.3 Emerging biomarkers

Clark et al. (136) utilized xCell to analyze the immune and stromal components of 103 ccRCC samples, integrating transcriptomic and proteomic data to classify ccRCC into four distinct subtypes: CD8^+^ inflamed tumors, CD8^−^ inflamed tumors, VEGF-high immune desert tumors, and metabolically active immune desert tumors. CD8^+^ inflamed tumors are characterized by extensive CD8^+^ T cell infiltration and elevated expression of inhibitory receptors such as PD-1, PD-L1, and CTLA-4, conferring poor prognosis but high potential for immunotherapy response. CD8^−^ inflamed tumors exhibit infiltration by CAFs and innate immune cells such as TAMs. VEGF-high immune desert tumors display pronounced vascularization due to elevated VEGF expression. Metabolically active immune desert tumors, with the lowest immune and stromal scores, exhibit upregulated expression of metabolic enzymes such as pyruvate kinase M (PKM) and peroxiredoxin-4 (PRDX4), along with activation of MYC and mTOR signaling pathways, indicative of tumor metabolic reprogramming. The Lung Immune Prognostic Index (LIPI) has recently emerged as a novel biomarker for immunotherapy, offering a valuable tool for risk stratification and personalized treatment decision-making across various malignancies. Initially applied in non-small cell lung cancer, melanoma, small cell lung cancer, head and neck squamous cell carcinoma, and bladder cancer, LIPI has also shown prognostic relevance in advanced RCC (145). Low-density lipoprotein receptor-related protein 6 (LRP6), a co-receptor in the Wnt/β-catenin signaling pathway involved in cell proliferation, inflammation, and transformation, has been correlated with drug sensitivity in clear cell RCC, suggesting its potential as a therapeutic target (146). Additionally, modulation of carcinoembryonic antigen-related cell adhesion molecule 1 (CEACAM1) signaling has been proposed as a novel approach in cancer immunotherapy. CEACAM1 expression is associated with disease progression, prognosis, and immune cell infiltration in clear cell RCC, highlighting its promise as both a predictive biomarker and a therapeutic target (147).

## 4 Immunotherapy

### 4.1 Immune checkpoint inhibitors

Preclinical studies have delineated the biological roles of PD-1, PD-L1, and CTLA-4, enabling clinical trials of ICIs for advanced RCC (148–150). CTLA-4, expressed on activated T cells, binds B7 molecules on antigen-presenting cells (APCs), inhibiting T cell activation (151). Ipilimumab, an anti-CTLA-4 antibody, restores T cell function by blocking CD80/CD86 interactions but has limited clinical utility due to a narrow therapeutic window (152). PD-1, another inhibitory checkpoint, binds PD-L1/PD-L2 on tumor cells, suppressing T cell activity. Nivolumab, a PD-1 inhibitor, showed superior OS and objective response rate (ORR) versus everolimus in the CheckMate-025 trial, leading to FDA approval for mRCC (150). Beyond PD-1/CTLA-4, other checkpoints like TIM-3, LAG-3, KIRs, and TIGIT modulate T cell function via distinct mechanisms, potentially compromising immunotherapy efficacy (153). Targeting these pathways is under clinical investigation in RCC (154). To enhance immunotherapy efficacy, clinical trials have investigated combining anti-PD-1/PD-L1 antibodies with anti-CTLA-4 antibodies or TKIs as first-line RCC treatments, demonstrating superior outcomes to TKI monotherapy (44). While both PD-1 and CTLA-4 inhibit T cell activation, CTLA-4 acts early in T cell priming, whereas PD-1 suppresses CD8^+^ T cell effector function in the TME (155). Dual blockade synergistically boosts CD8^+^ T cell activation and accumulation (156). The CheckMate-214 trial showed ipilimumab-nivolumab improved PFS, ORR, and OS in intermediate-/high-risk RCC versus sunitinib, leading to its approval for these patients (157).

In breast cancer models, ICIs activate CD8^+^ T cells, inducing tumor vessel normalization, which alleviates TME immunosuppression, enhancing T cell infiltration and cytotoxicity—a positive feedback loop underpinning ICI combinations (158). The KEYNOTE-426 trial reported pembrolizumab-axitinib outperformed sunitinib across risk groups and PD-L1 levels (159), while JAVELIN Renal-101 showed avelumab-axitinib improved PFS by 6.6 months versus axitinib alone (160). These results led to FDA approval of both ICI-TKI regimens. The CLEAR study revealed lenvatinib-pembrolizumab provided durable survival benefits over sunitinib (160). Despite their frontline status, combination therapies are not universally effective and may cause severe toxicity. In KEYNOTE-426, pembrolizumab-axitinib frequently induced diarrhea, hypertension, and hepatic toxicity, with 30.5% discontinuing at least one drug due to adverse events (159). Biomarker-driven patient stratification is crucial to mitigate toxicity and costs, alongside deeper investigation of drug interactions to guide monotherapy or sequential approaches when appropriate.

### 4.2 Targeting immunosuppressive cells

**Figure 1:**
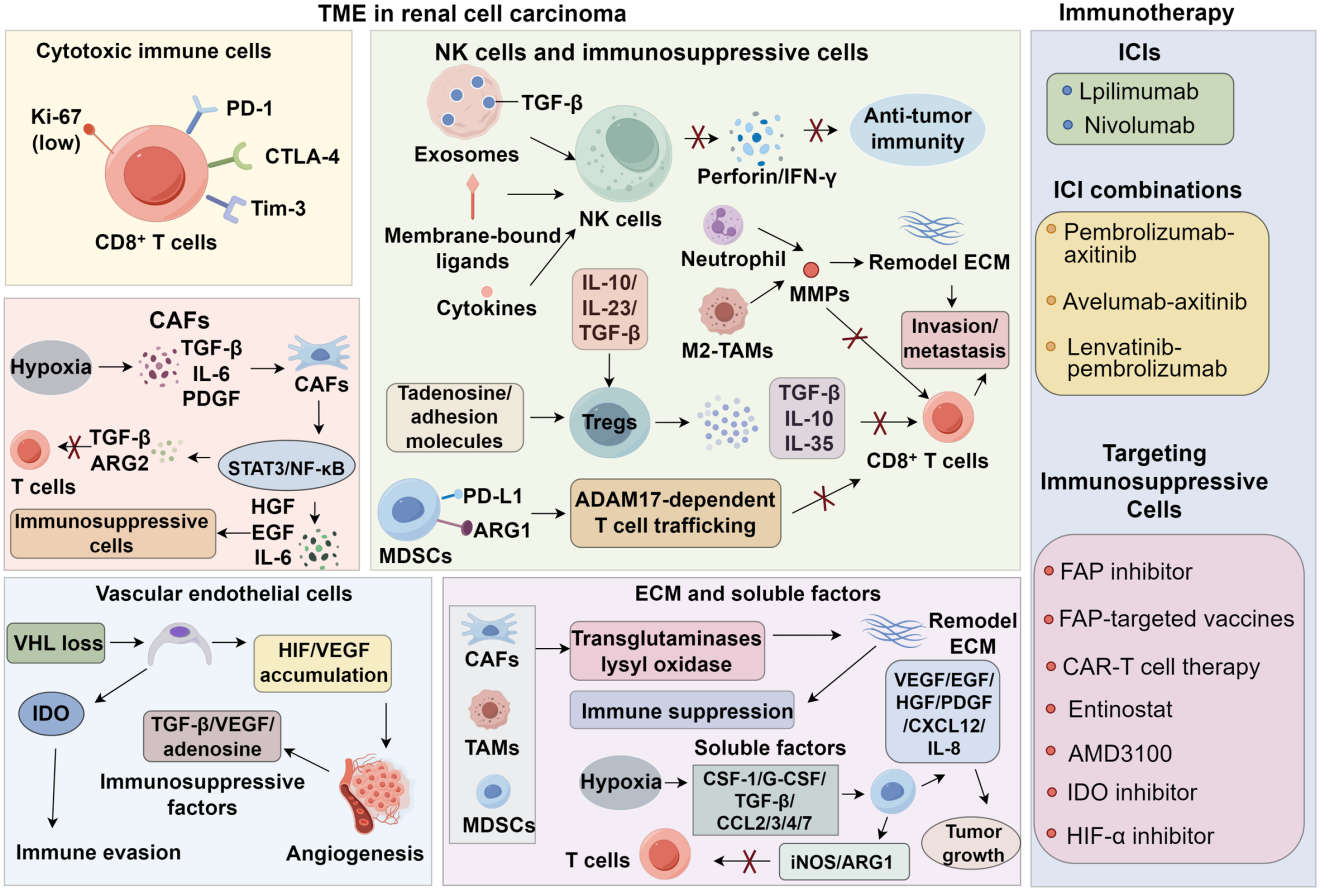
Tumor microenvironment and potential immunotherapy strategies in renal cell carcinoma.

Current therapeutic strategies targeting immunosuppressive cells in the RCC TME can be broadly categorized into three types (161). The first strategy involves depleting immunosuppressive cells to restore CD8^+^ T cell infiltration and enhance anti-tumor immunity. Fibroblast activation protein (FAP), a surface marker broadly expressed by CAFs in epithelial tumors, is a strong predictor of tumor invasiveness. Agents that inhibit FAP activity, anti-FAP antibodies, FAP-targeted vaccines, and CAR-T cell therapy have shown efficacy in depleting CAFs in preclinical models of malignancies such as mesothelioma (162). The second strategy aims to normalize immunosuppressive cells by inducing CAF quiescence, promoting MDSC maturation, or repolarizing M2-type TAMs. In murine RCC models, entinostat suppressed the immunosuppressive activity of MDSCs by inhibiting ARG1 and iNOS, thereby enhancing CD8^+^ T cell infiltration (16). The combination of entinostat with atezolizumab and bevacizumab is currently being tested in clinical trials for advanced RCC (NCT03024437). The third strategy focuses on modulating downstream pathways of immunosuppressive cells. The CXCR4–CXCL12 axis plays a critical role in the recruitment of MDSCs and Tregs to the RCC TME. The CXCR4 antagonist AMD3100 has been shown to impair the immunosuppressive function of these cells and improve anti-tumor immune responses (163). Given the frequent occurrence of mutations in metabolism-related genes, RCC is also considered a metabolic disease. Metabolic reprogramming in RCC involves aerobic glycolysis, fatty acid metabolism, and the utilization of tryptophan, glutamine, and arginine, enabling tumor cells to adapt to hypoxia and nutrient depletion while evading immune surveillance (164). IDO contributes to local tryptophan depletion in the TME via the kynurenine pathway, leading to T cell exhaustion and apoptosis. Thus, IDO inhibition can relieve local immune suppression and enhance T cell activity (165). A phase I/II clinical trial is currently evaluating the combination of the IDO inhibitor epacadostat with the anti-PD-1 antibody pembrolizumab in various solid tumors, including RCC, with promising results previously reported in melanoma (166–168). Inhibitors of HIF-α and glutaminase have also entered clinical trials for RCC (169, 170) (**Figure 1**).

### 4.3 Clinical strategies to overcome resistance and manage toxicity

The clinical application of immunotherapy in RCC is constrained by tumor heterogeneity, acquired resistance, and treatment-related toxicity, and overcoming these challenges requires an integrated approach (171). Recent progress emphasizes adaptive and biomarker-driven trial designs which stratify patients according to PD−L1 expression, PBRM1 mutation status, or immune subtype to achieve precision therapy (172–174). Another key strategy is sequencing therapy rather than administering agents concurrently; for example, initiating treatment with TKIs to normalize aberrant vasculature and subsequently introducing ICIs can enhance immune cell infiltration while reducing overlapping toxicities (175). Efforts to counteract resistance also include the incorporation of novel agents such as TAM-reprogramming compounds, selective HIF−2α inhibitors, and metabolic modulators into combination regimens to disrupt pro-tumorigenic pathways (176, 177). Equally important is the proactive management of immune-related adverse events, which relies on early recognition, multidisciplinary collaboration, and the use of standardized treatment algorithms with corticosteroids or selective immunosuppressants to preserve antitumor activity (178, 179). Together, these strategies are shaping current and future clinical trials and provide clinicians with practical guidance to optimize therapeutic outcomes while minimizing toxicity in patients with RCC.

## 5 Conclusion

The immunosuppressive TME of RCC remains a major barrier to durable therapeutic responses, despite significant progress in immunotherapy. The interplay between cytotoxic immune cells and immunosuppressive components creates a permissive niche for tumor progression. While ICIs and combination therapies have revolutionized treatment, their efficacy is limited by intrinsic and acquired resistance, as well as toxicity. Biomarkers such as PD-L1, TMB, and PBRM1 mutations offer predictive insights but lack universal applicability, highlighting the need for multi-parametric profiling. Emerging strategies, including TAM repolarization, CAF depletion, metabolic modulation, and targeting novel immune checkpoints, hold promise but require further validation in clinical trials.

Looking ahead, advanced technologies will be pivotal in overcoming these limitations. Single-cell multi-omics and spatial transcriptomics enable high-resolution mapping of cellular states, lineage trajectories, and intercellular communication within the RCC TME, providing insights that bulk analyses cannot capture. These approaches will help to identify novel cellular subsets, spatially restricted immunosuppressive niches, and potential therapeutic targets. Additionally, artificial intelligence and machine learning are increasingly being applied to integrate multi-dimensional datasets, including genomics, transcriptomics, imaging, and clinical data, to develop predictive models for patient stratification and to discover novel biomarkers. Together, these emerging technologies hold great promise for bridging existing knowledge gaps, enabling real-time monitoring of TME evolution, and guiding the development of precision immunotherapies tailored to individual RCC patients. Moving forward, integrating these innovations with multi-omics profiling and optimizing treatment sequencing will be critical to overcoming resistance and improving outcomes. Ultimately, a precision medicine approach, guided by TME dynamics and predictive biomarkers, will be essential to unlocking the full potential of immunotherapy in RCC.
